# Supplementary material for: Matrin 3-dependent neurotoxicity is modified by nucleic acid binding and nucleocytoplasmic localization
Source: eLife. 2018 Jul 17;7:e35977. doi: 10.7554/eLife.35977 (PMC6050042; doi:10.7554/eLife.35977)
Supplement: Transparent reporting form [file elife-35977-transrepform.docx]

***eLife’s* transparent reporting form**

We encourage authors to provide detailed information *within their submission* to facilitate the interpretation and replication of experiments. Authors can upload supporting documentation to indicate the use of appropriate reporting guidelines for health-related research (see [EQUATOR Network](http://www.equator-network.org/%20)), life science research (see the [BioSharing Information Resource](https://biosharing.org/)), or the [ARRIVE guidelines](http://www.plosbiology.org/article/info:doi/10.1371/journal.pbio.1000412) for reporting work involving animal research. Where applicable, authors should refer to any relevant reporting standards documents in this form.

If you have any questions, please consult our Journal Policies and/or contact us: [editorial@elifesciences.org](mailto:editorial@elifesciences.org).

**Sample-size estimation**

- You should state whether an appropriate sample size was computed when the study was being designed
- You should state the statistical method of sample size computation and any required assumptions
- If no explicit power analysis was used, you should describe how you decided what sample (replicate) size (number) to use

Please outline where this information can be found within the submission (e.g., sections or figure legends), or explain why this information doesn’t apply to your submission:

Based on crude estimates of sample size (described in Chow S, Shao J, Wang H. 2008, *Sample Size Calculations in Clinical Research*, 2nd Ed. Chapman & Hall/CRC Biostatistics Series), we determined that we would need to count approximately 200 neurons per sample to distinguish a 20% difference in survival with 80% power and 95% confidence. Most counts significantly exceed this threshold. To enhance confidence further, all studies involved a minimum of 8 technical replicates per condition, and a minimum of 3 biological replicates.

**Replicates**

- You should report how often each experiment was performed
- You should include a definition of biological versus technical replication
- The data obtained should be provided and sufficient information should be provided to indicate the number of independent biological and/or technical replicates
- If you encountered any outliers, you should describe how these were handled
- Criteria for exclusion/inclusion of data should be clearly stated
- High-throughput sequence data should be uploaded before submission, with a private link for reviewers provided (these are available from both GEO and ArrayExpress)

Please outline where this information can be found within the submission (e.g., sections or figure legends), or explain why this information doesn’t apply to your submission:

Technical replicates refer to individual wells of cultured neurons within a 96-well plate. Biological replicates include separate cultures of neurons isolated from different pregnant dams. We used 8 technical replicates per condition to capture and account for variability among different wells. The number of biological replicates varied depending on the experiment. Data in Figure 1 are pooled from 3 biological replicates, Figure 2B from >3 biological replicates, Figure 2D-E data from 4 biological replicates, Figure 3C from 4 biological replicates, Figure 3E from 5 biological replicates, Figure 4 from 3 biological replicates, Figure 6E from 5 biological replicates, Figure 6F from 3 biological replicates, and Figure 7C-D from 3 biological replicates. All Western blot experiments involved 3 biological replicates. Exact sample sizes can be found in figure legends.

**Statistical reporting**

- Statistical analysis methods should be described and justified
- Raw data should be presented in figures whenever informative to do so (typically when N per group is less than 10)
- For each experiment, you should identify the statistical tests used, exact values of N, definitions of center, methods of multiple test correction, and dispersion and precision measures (e.g., mean, median, SD, SEM, confidence intervals; and, for the major substantive results, a measure of effect size (e.g., Pearson's r, Cohen's d)
- Report exact p-values wherever possible alongside the summary statistics and 95% confidence intervals. These should be reported for all key questions and not only when the p-value is less than 0.05.

Please outline where this information can be found within the submission (e.g., sections or figure legends), or explain why this information doesn’t apply to your submission:

Statistical tests used, sample sizes, and exact p-values are in the figure legends following each panel description.

(For large datasets, or papers with a very large number of statistical tests, you may upload a single table file with tests, Ns, etc., with reference to sections in the manuscript.)

**Group allocation**

- Indicate how samples were allocated into experimental groups (in the case of clinical studies, please specify allocation to treatment method); if randomization was used, please also state if restricted randomization was applied
- Indicate if masking was used during group allocation, data collection and/or data analysis

Please outline where this information can be found within the submission (e.g., sections or figure legends), or explain why this information doesn’t apply to your submission:

All analyses of survival data in Figures 1, 2B, 2D-E, 3C, 3E, 4, 6E, 6F, and 7C-D were performed in an unbiased and fully automated fashion, thereby removing the possibility of bias. All remaining analyses of biochemical or image-based data were performed in a blinded fashion using ImageJ.

**Additional data files (“source data”)**

- We encourage you to upload relevant additional data files, such as numerical data that are represented as a graph in a figure, or as a summary table
- Where provided, these should be in the most useful format, and they can be uploaded as “Source data” files linked to a main figure or table
- Include model definition files including the full list of parameters used
- Include code used for data analysis (e.g., R, MatLab)
- Avoid stating that data files are “available upon request”

Please indicate the figures or tables for which source data files have been provided:

N/A
